# Radiation therapy in primary orbital lymphoma: a single institution retrospective analysis

**DOI:** 10.1186/1748-717X-4-60

**Published:** 2009-12-07

**Authors:** Luigi De Cicco, Laura Cella, Raffaele Liuzzi, Raffaele Solla, Antonio Farella, Giorgio Punzo, Fausto Tranfa, Diego Strianese, Manuel Conson, Giulio Bonavolontà, Marco Salvatore, Roberto Pacelli

**Affiliations:** 1 Department of Diagnostic Imaging and Radiation Oncology, University "Federico II" of Naples, Italy; 2 Institute of Biostructures and Bioimages, National Council of Research (CNR), Naples, Italy; 3 Department of Orbital Pathology, University "Federico II" of Naples, Italy

## Abstract

**Background:**

Primary orbital lymphoma is a rare disease that accounts for 10% of all orbital tumors. Radiotherapy on the orbital cavity is the treatment of choice for this unusual presentation of localized non-Hodgkin's lymphoma (NHL). The aim of this study is to retrospectively evaluate the effectiveness and the toxicity of radiation treatment in patients with primary orbital lymphoma.

**Methods:**

Forty-seven consecutive patients having primary orbital lymphoma treated in our department between May 1983 and September 2006 were investigated in a retrospective study. Either ^60^Co γ rays or 6 MV X rays were used to deliver daily fractions of 1.8 or 2.0 Gy, 5 times/week, with total doses ranging from 34.2 to 50 Gy. Forty-three patients had stage IE, three had stage II and one stage IV disease. Thirty-eight patients had marginal zone B-cell lymphoma, 5 diffuse large B cell lymphoma, 3 mantle cell lymphoma and 1 Burkitt lymphoma. Local control (LC), disease free survival (DFS), overall survival (OS) and late side effects were evaluated in all patients.

**Results:**

With a median follow up of 45 months, LC was obtained in 100% of patients. The estimated 5- and 7-year DFS rates were 75.8% and 55.3%, and the 5- and 7-year OS rates were 88.7% and 79.9% respectively. Acute toxicity was minimal. Late toxicity such as cataract, keratitis, retinopathy and xerophthalmia occurred respectively in 12 (25.5%), 5 (10.6%), 1 (2.1%), and 9 (19.1%) patients.

**Conclusion:**

Radiotherapy is an effective and at the same time well tolerated treatment for primary orbital lymphoma.

## Background

The orbit is a rare site of presentation of non-Hodgkin's lymphoma (NHL). Primary orbit lymphoma (POL) represents 1% of all NHLs and 8% of extranodal NHLs [1]. Bilateral involvement occurs in 10-15% of cases of POL [2]. The majority of patients at the time of diagnosis are over 65 [3,4]. The commonest manifestation of the disease is a slowly growing orbital mass that can be either asymptomatic, or, depending on the location of the tumor, associated with proptosis, ocular dysmotility, periorbital swelling, blurring of vision and chemosis. The most frequent histology of POL is indolent NHL such as extranodal marginal B-cell lymphoma of mucosa-associated lymphoid tissue (MALT) [5-8]. Usually therapeutic management of POL consists of radiation treatment [6,8-25] encompassing the entire orbit [26]. At present it is known that radiotherapy, using low or moderate doses in the range of 25-36 Gy, can obtain 95-100% of local control. However, due to the rarity of the disease, data about doses from comparative studies are not available and, moreover, in reports with a substantial number of treated patients, doses in the range of 20 and 57 Gy were found to be used [6,8-25]. For patients with advanced disease, sequential chemo-radiation treatment is preferred [8].

In this study we have retrospectively evaluated the effectiveness and late toxicity of radiation therapy in 47 consecutive patients with a diagnosis of POL treated at the Radiation Oncology Department of the Medical School of the University "Federico II" of Naples. In particular, we evaluated our treatment with respect to local control (LC), disease free survival (DFS), overall survival (OS), and incidence of late side effects such as cataract, keratitis and xerophthalmia.

## Methods

### Patient section

From May 1983 to September 2006, 47 patients (and 49 orbits) affected with primary biopsy-proven orbital NHL were treated at the Radiotherapy Department of University "Federico II" of Naples. Patient's median age at diagnosis was 62 years (range 33-88 years). The vast majority (38 out of 47) of patients had extranodal marginal zone B-cell lymphoma of mucosa-associated limphoid tissue (MALT) type NHL according to REAL classification [27]. All patients had medical history and physical examination and underwent orbits CT scan and bone marrow biopsy. Thirty four (72.3%) patients underwent further staging by total body CT scan, 11 (23.4%) by total body ^18^FDG-PET scan, and 2 (4.3%) by chest X-ray. Forty three out of 47 patients resulted to have stage IE, 3 patients had stage II (2 bilateral orbital involvement and 1 ipsilateral parotid involvement) and 1 stage IV disease (bone marrow involvement) according to Ann-Arbor classification. Detailed description of patient features is reported in table 1.

**Table 1:** Patients' clinical features.

Patients	N.	%
*Gender*		
Male	20	42.6
Female	27	57.4

Localization*		
Orbital cavity	41	83.7
Lachrymal sac	1	2.0
Conjunctiva	3	6.1
Eyelid	4	8.2

*Histology*		
B cell MALT lymphoma	38	80.9
Diffuse large B cell lymphoma	5	10.6
Mantle cell lymphoma	3	6.4
Burkitt lymphoma	1	2.1

*Stage*		
I	43	91.5
II	3	6.4
III	0	0
IV	1	2.1

* Total number of localizations are 49 since two patients had bilateral orbital involvement

### Treatment

Radiation treatment was delivered with total doses ranging from 34.2 to 50 Gy with a median dose of 36 Gy administered by daily fractions of 1.8-2.0 Gy, 5 times/week. From 1981 to 1998, the treatment was administered by Co-60-gamma-Ray (14 patients), and since 1999 the treatment was administered with 6 MV X photons from a LINAC (33 patients). For 43 patients, a direct anterior field was used, while an anterior plus a lateral field was used for the remaining 4 patients. The whole orbital cavity was included in the target. For the patient with parotid involvement, we used a separate additional electron beam lateral field encompassing the whole gland. In all patients treated by LINAC, a CT based simulation software (Computerized Medical System, Inc., St Louis, MO) for target and lens contouring was used and treatment planning was performed by a 3-D planning system (XiO 4.4, Computerized Medical System, Inc., St Louis, MO). Lens shielding was performed for the treatment of 38 (77.6%) orbits in which tumour coverage was not compromised and the site of disease was not eyelid or conjunctiva. For the anterior field, lens shielding was obtained using eye customized shielding block placed on the blocking tray over the lens, and checked daily by the physician. When the lateral field was used, the anterior border was always placed posterior to the lens. Superficial lesions with involvement of conjunctiva or eyelid were treated without lens shielding and with bolus to bring the isodose curve to surface for adequate coverage. Details on prescribed radiation doses are reported in table 2.

**Table 2:** Radiation doses. Fraction dose 1.8 - 2.0 Gy.

Total dose (Gy)	Patient N. (%)
34.2	1 (2.1)
36	28 (59.6)
40	6 (12.8)
41.4	1 (2.1)
42	1 (2.1)
44	5 (10.6)
45	1 (2.1)
46	1 (2.1)
50	3 (6.4)

Two patients received systemic chemotherapy: one patient, with aggressive Burkitt Stage I orbital lymphoma, underwent concomitant chemo-radiation therapy; the other one, with Stage IV disease, had first chemotherapy, then radiotherapy on the residual orbital mass.

Acute and late ocular side effects were evaluated and graded according to RTOG toxicity score.

The follow-up consisted in patient's history, physical examination, ophthalmologist evaluation every 4 months and an imaging study of the orbits that included either CT or MRI or ultrasonography scan. Total body CT scan was prescribed yearly for 5 years.

### Statistical Analysis

Categorical data were expressed as percentage. Chi-square analysis with Yates correction and Fisher's exact test were applied when appropriate; a p-value of 0.05 was chosen as significant.

Local Control (LC), disease free survival (DFS) and overall survival (OS) were analyzed statistically in all patients. DFS was calculated from the date of the end of the radiation therapy to the date of first documented relapse (event). A patient dead for any other cause or a patient lost to follow-up for reasons unrelated to the study was considered a censored observation. OS was calculated from the date of the end of the radiation therapy to the date of death or the date of the last follow up. The Kaplan-Meier method was used to estimate the rates of DFS and OS. Statistical evaluation was carried out using SPSS 13.0 statistical software.

## Results

At the first control after radiotherapy, all patients resulted free of disease. With a median follow up of 45 months (range 5-203) no local relapses were observed and the LC resulted to be 100%.

Twelve (25.5%) patients failed distantly with a median time for failure of 44 months: two patients relapsed in the other eye, 4 at ipsilateral laterocervical nodes, 3 in the abdomen, 1 in the mediastinum, 1 in the trunk skin, and 1 in a vertebral bone. All relapsed patients but one had undergone total body CT scan at the time of diagnosis.

Eight out of 43 (18.6%) Stage I patients failed distantly, while 4 out of 4 (100%) Stage II-IV patients failed distantly (p = 0.003). Eight out of 38 patients with MALT type lymphoma failed distantly and 4 out of 9 patients with different histotype failed distantly (p = 0.15).

**Figure 1:**
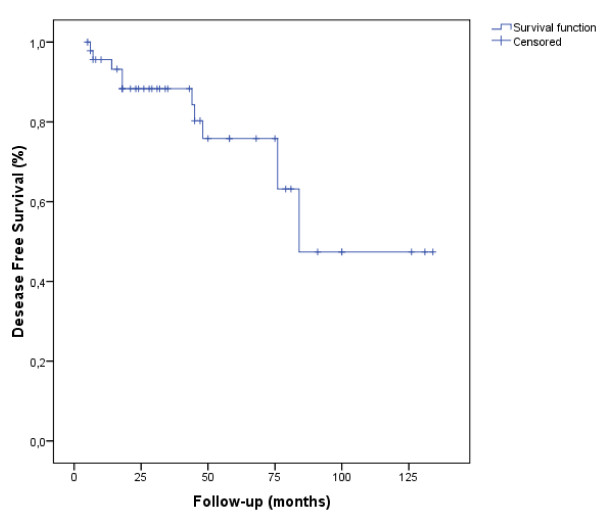
**Disease-Free Survival (DFS)**. The delay of DFS was calculated from the date of the end of radiotherapy until the date of revealing of a progress or until the date of death, or until the date of last news (47 patients, 12 events, 35 censored).

**Figure 2:**
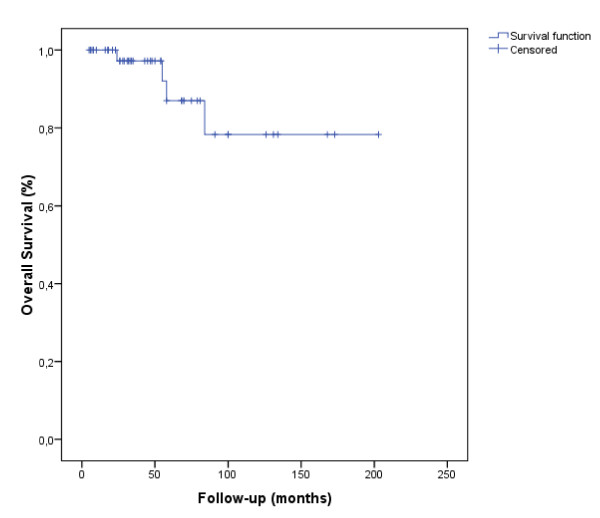
**Overall survival (OS)**. The delay of OS was calculated from the date of the end of radiotherapy until the patient's death or until the date of last news (47 patients, 4 events, 43 censored).

The 5- and 7-year DFS rates were 75.8% and 55.3% (Figure. 1), respectively and the 5- and 7-year OS rates were 88.7%and 79.9% (Figure. 2).

All the relapsed patients underwent second line therapy (radio or chemotherapy). Nine are presently free of disease, 2 are alive with disease, and 1 is death for lymphoma (Cause Specific Survival was 97.9%). Four patients (8.5%) died from non lymphoma-related causes.

None of the patients developed significant acute toxicity; the most frequent side effects were mild conjunctivitis, excessive tearing or dryness, and periorbital erythema or oedema. According to RTOG acute toxicity score, twenty-three (48.9%) patients had G1 toxicity and only 2 (4.3%) had G2 toxicity and required artificial tears during radiation treatment.

As a whole, no G3-4 RTOG late side effects were reported in any patient. Keratitis occurred in 5 patients (10.6%), xerophthalmia in 9 (19.1%), cataract in 12 (25.5%), corneal ulceration occurred in one patient (2.1%), and minor retinopathy in one (2.1%).

Cataract, minor retinopathy and corneal ulceration occurred in 20.7% patients among those treated with a dose ≤ 36 Gy (6 on 29 patients), and in 44.4% patients among those treated with doses > 36 Gy (8 on 18 patients) (p = 0.083).

A lens-sparing technique was used in all patients with the exception of 9 patients for which lens shielding was not used because tumour coverage could be compromised. Three of 9 patients (33%) without lens shielding developed cataract, while 9 of 38 patients (23%) with lens shielding developed cataract (p = 0.31).

## Discussion

In POL bearing patients, a complete staging evaluation is necessary at first diagnosis for treatment decision [10,18]. Exclusive surgical approach is not recommended because of the high rate of local relapses [23], probably as a result of the inherent difficulty of preserving ocular function and encompassing all local disease; so surgery is limited to biopsy. Esik *et al*., comparing different modalities of treatment for orbital NHL, showed a 10-year local control of 0% in the group of patients treated with surgery only. Although observation [28] or first line chemotherapy [29,30] have been recently proposed and sequential chemo-radiotherapy is more judicious for treating intermediate/high grade lymphomas [11] and primary systemic disease [8], at present radiotherapy is the treatment of choice for NHL localized in the orbital cavity. According to literature [6,8-25], it is possible to obtain local control in the range of 89-100% using radiotherapy in low or moderate doses as 25-36 Gy. A summary of findings of some of published major studies on the issue is shown in table 3.

**Table 3:** Review of radiotherapy studies on orbital lymphoma.

Series	N° of patients	Stage	Histology	Dose (Gy)	Local control	Survival at 5 years
Jereb et al.[3]	19	I, II and IV	Low and intermediate	20-37.5	100%	NA
Smitt et al.[9]	25	I-II	Low, intermediate and high	28-40.2	89%	93%
Chao et al.[10]	20	I	Low and intermediate	20-43.2	100%	90%
Bolek et al[11]	20	I-II	Low, intermediate and high	15-53.3	95%	Low 89%, interm and high 33%
Stafford et al[13]	48	I-II	Low, intermediate and high	15-53.8	98%	88%
Liao et al.[32]	25	I	Low and intermediate	30-40	100%	NA
Bhatia et al.[15]	47	I	Low, intermediate and high	20-51	100%	74%
Martinet et al.[18]	90	I-II	Low, intermediate and high	4.0-50.4	97%	78%
Fung et al. [6]	98	I and III-IV	MALT, Follicular DLBCL	16.2-46	98%	Stage I,94% III-IV, 49%
Zhou et al. [20]	46	I-IV	Low, intermediate and high	30.6	98%	88%
Aviles et al.[33]	98	IE	MALT	34-40	98%	96%
Bischof et al. [21]	42	I-IV	Low, intermediate and high	20-46	80-100%	Stage I, 91% II, 80% III-IV, 47%
Nam H et al.[34]	66	I	MALT	20-45	97%	NA
Son SH [35]	46	IE	MALT	21.6-45	98%	100%

DLBCL: diffuse large B-cell lymphoma, MALT: mucosa associated lymphoid tissue, NA: not assessed

Recently, in a review of the literature on radiation treatment of POL, Yadav *et al*. report about acute and late toxicity of this type of therapeutic modality. The most frequent acute side effect is conjunctivitis that should be treated with artificial tears, while the most frequent late toxicity is cataract that is today treated in a safe and efficient way by surgical technique [31].

In our series, a median treatment dose of 36 Gy was used to treat patients affected with POL. At a median follow-up of 45 months local control rate of 100% was obtained. Eight out of 43 (18.6%) Stage I patients failed distantly, while 4 out of 4 (100%) Stage II-IV patients failed distantly. Disease stage at diagnosis influenced the systemic relapse incidence (p = 0.003), while histologic grade did not significantly influence outcome, maybe also due to the low number of patients compared. Some studies suggested a dose of 36-40 Gy for high-grade orbital NHL [11,23]. In our series, radiation doses for non MALT type NHL were slightly greater than doses used for MALT type disease, with a median of 40 Gy. However, given the excellent local control, no dose relation was found at the outcome.

Prognostic value of age has been reported in POL, showing that patients older than 64 year fared worse than younger patients [18]. In the present study we have stratified patients according to the age into two groups, one of patients older than 64 year and the other group including the younger patients. However, no differences in DFS (p = 0.73) were found.

Considering late side effects, cataract, minor retinopathy and corneal ulceration developed in 30% of patients (14 out of 47). As expected, toxicity was more frequent in patients receiving more than 36 Gy, although the difference did not reach statistical significance (p = 0.083), probably due to the low number of events. Nevertheless, other treatment-independent risk factors for the process of cataract development like diabetes mellitus, drugs, familiar predisposition and age cannot be excluded. As a whole, the incidence and the severity of acute and late toxicity were acceptable and consistent with other reports [18,31].

Our data, according to literature, support radiation therapy as principal treatment modality in orbital localization of NHL and suggest that the optimal dose to achieve both disease control and minimum late effects has not to be greater than 36 Gy. At present the dose used in our department is 30 Gy as recommended by current literature.

## Competing interests

The authors declare that they have no competing interests.

## Authors' contributions

LDC, LC and RP conceived and designed the study. LC, RL, RS, AF, GP, FT, DS, MC, GB, and RP treated patients on the study. RP, MS, RL and LC analyzed the data. All authors participated in drafting and revising the manuscript. All authors have given final approval of the manuscript.
